# The longitudinal effect of clozapine-associated sedation on motivation in schizophrenia

**DOI:** 10.1192/bjp.2022.191

**Published:** 2023-07

**Authors:** Noham Wolpe, Shanquan Chen, Brian Kirkpatrick, Peter B Jones, Christopher Jenkins, Rudolf N. Cardinal, Emilio Fernandez-Egea

**Affiliations:** 1 Department of Physical Therapy, The Stanley Steyer School of Health Professions, Faculty of Medicine, Tel Aviv University, Tel Aviv 6997801, Israel; 2 Sagol School of Neuroscience, Tel Aviv University, Tel Aviv 6997801, Israel; 3 Department of Psychiatry, University of Cambridge, Cambridge CB2 0SZ, UK; 4 Cambridgeshire and Peterborough NHS Foundation Trust, Fulbourn, Cambridge CB21 5EF, UK; 5 Psychiatric Research Institute, University of Arkansas for Medical Sciences, Little Rock, Arkansas, USA

## Abstract

Negative symptoms of schizophrenia manifest as reduced motivation and pleasure (MAP) and impaired emotional expressivity (EXP). These can occur as primary phenomena, but have also been suggested to occur secondary to other clinical factors, including antipsychotic-induced sedation. However, this relationship has not been established formally. Here, we examined the effect of antipsychotic-induced sedation (assessed via the proxy of total daily sleep duration) on MAP and EXP in a cohort of 187 clozapine-treated patients with schizophrenia followed for over two years on average, using multi-level regression and mediation models. MAP, but not EXP, was adversely influenced by sedation, independently of the severity of psychosis or depression. Moreover, clozapine impaired MAP indirectly by worsening sedation, but after accounting for clozapine-induced sedation, clozapine improved MAP within patients. Our results highlight the importance of addressing sedative side-effects of antipsychotics to improve clinical outcomes.

## Introduction

Although ‘negative’ symptoms of schizophrenia play a central role in long-term outcome^1^, they remain poorly treated in clinical practice, where treatment is typically centred on minimising ‘positive’ (psychotic) symptoms. Once considered a single construct, recent work has described five distinct negative symptom domains, namely avolition, asociality, anhedonia, alogia (or poverty of speech), and blunted affect^2^. Newer clinical assessment tools have been designed to cover these five principal domains, including the Brief Negative Symptoms Scale (BNSS)^3^. Latent structure analyses have reduced the five symptom domains into two main clinical factors: (1) impaired motivation and pleasure (MAP), comprising avolition, asociality, and anhedonia; and (2) emotional expressivity deficits (EXP)^3^, comprising alogia and blunted affect (but see^2^). Negative symptoms can also be classified as primary and secondary. While primary negative symptoms are intrinsic to schizophrenia, secondary negative symptoms are caused by medication side effects, psychosis, depression, substance misuse and social deprivation^1^. This latter distinction is relevant clinically, as there is currently no treatment for primary negative symptoms, while secondary negative symptoms are potentially treatable^1^.

Antipsychotic-induced akinesia and sedation are cited as a source for secondary negative symptoms, but there is only weak anecdotal evidence for this association^4^. We investigated the longitudinal effect of antipsychotic-induced sedation on negative symptoms in a well-characterised cohort of patients with schizophrenia treated with clozapine, one of the most sedating antipsychotics^5^. Patients were assessed for sedation (assessed using total hours of sleep per day^6^) and for negative symptoms using the BNSS^3^. We addressed three questions: 1) Does sedation influence negative symptoms, and, if so, does it affect MAP or EXP? 2) Is the effect independent of other confounders? 3) What is the direct and indirect impact of clozapine on MAP? We hypothesised that drug-induced sedation would specifically impair MAP, even after controlling for other clinical variables.

## Methods

### Study design and participants

This was a naturalistic longitudinal study of clozapine-treated patients attending Cambridgeshire and Peterborough NHS Foundation Trust, UK. The cohort has been well characterised for sociodemographic and clinical information, including via the Positive and Negative Syndrome Scale (PANSS) and depression measured through the Depression Scale for Schizophrenia (CDSS)^7^. The cohort has been described in detail elsewhere^6^ (see Supplementary Methods).

### Assessments of sedation and negative symptoms

As a proxy for sedation, we used the total number of hours of sleep per day (overall daytime and night-time sleep). We have previously shown that this measure provides a reliable estimate of antipsychotic induced sedation^6^. As in our previous work, we corroborated the self-reported total number of hours of sleep through additional questions about sleep habits ^6^ (Supplementary Methods).

Negative symptoms were assessed using the 13-item BNSS^3^. The Lack of Normal Distress item (item 4) was not considered, as it is not typically included in the analysis of negative symptoms^3^. We used the two main clinical factors of MAP (sum of items 1−3 and 5−8) and EXP (sum of items 9−13)^3^. For readability, we reversed the score for each domain severity to 6=normal, and 0=impaired, such that when added up to calculate MAP and EXP severity, increased scores more intuitively reflected improved clinical presentation (more motivation and more emotional expression, respectively).

### Statistical analyses

The association between sedation and the severity of negative symptoms was assessed using multi-level regression models. We ran two separate models, with MAP and EXP as the dependent variables. A random-effects intercept was fitted for each participant, and the main (fixed slope) variable was sedation. We covaried for age at baseline, sex, positive symptoms, depression, clozapine dose, smoking (categorical variable: yes vs. no), number of units of alcohol consumed per week (1 UK unit = 10 ml), and aripiprazole dose. Aripiprazole was included as we have previously shown the independent effect of aripiprazole to reduce sedation^6^ and because it is the most common augmentation medication in this cohort, and thus could be reliably modelled statistically.

Finally, a multi-level mediation model was used to assess the longitudinal effect of clozapine on motivation, both directly and indirectly via its effect on sedation. We included the same set of covariates as above. Coefficients, their 95% confident intervals (CIs), and *p* values were estimated for each path and for the total direct and indirect paths, with a random-effect intercept for each participant.

All statistical analyses were performed using R (version 3.5.0), with the lmerTest (version 3.1-2) and Mediation (version 4.5.0) packages.

## Results

A total of 187 clozapine-treated individuals were included, with 398 face-to-face assessments (mean follow-up period of 25 months). Clinical and sociodemographic data are shown in Table S1 and Table S2. All patients were on clozapine for at least one year at baseline assessment.

We found a significant association between the level of sedation and MAP longitudinally (*β*=-0.57, *p*=0.039), such that increased sedation levels were linked to reduced MAP levels within subjects. In addition to sedation, the severity of psychosis and depression (other known causes of secondary negative symptoms) were associated with a significant worsening of MAP (Table S3). By contrast, there was no such association between sedation levels and EXP (*β*=-0.19, *p*=0.391). For completeness^2^, exploratory analyses on the association between sedation levels and each negative symptom domain can be found in the Supplementary Material (Fig. S2).

To explore the specific effect of clozapine-induced sedation on MAP, we performed a longitudinal multi-level mediation analysis (Fig. 1B). This allowed us to separate, statistically, the clozapine-related sedation component and test its effect on MAP within subjects, while still controlling for other potentially confounding clinical variables. We found a significant effect of clozapine-related sedation on MAP (*β*=-0.0011, *p*=0.046), suggesting that clozapine-related sedation worsened MAP within subjects. However, after accounting for the clozapine-related sedation, clozapine improved MAP within subjects (*β*=0.0087, *p*=0.042).

## Discussion

We found that sedation (measured through the proxy of total sleep duration) impaired MAP, but did not affect EXP, as measured longitudinally through the BNSS. The effect of sedation on MAP was independent of other sources of secondary negative symptoms, such as psychosis or depression. To our knowledge, our study provides the first data-supported link between sedation and secondary negative symptoms, and specifically MAP. While interpreting our results, the main limitations of our analyses should be considered, namely the use of a single clinical rater and the need for larger sample sizes to ask more specific questions about other clinical modifiers (see Supplementary Discussion).

A major strength of our study comes from the use of longitudinal mediation analysis for measuring the direct and indirect effect of an antipsychotic medication on negative symptoms. This method allowed to explore the direction of the association of clozapine dose with motivation, both through its sedative effect (indirect) and after accounting for clozapine-related sedation (direct)—all while controlling for potential confounders. We found evidence that clozapine directly improves motivation, but this is opposed by the negative (but smaller) effect of clozapine-induced sedation on motivation. This result is consistent with earlier findings suggesting that clozapine improves negative symptoms^8^, but there is contradictory evidence in the literature^9^. A potential explanation for these mixed results is that previous research did not dissect the different (and opposite) effects of clozapine on sedation and motivation, as we did in this study. These results argue for the optimisation of clozapine dose to minimise secondary negative symptoms when treating psychosis in schizophrenia, so as to improve long-term clinical outcome in patients.

Unlike the effect on MAP, there was no association between sedation and EXP. This distinct effect on a specific negative symptom factor raises the intriguing speculation that there are distinct modulators and thus potential treatments for MAP and EXP deficits in schizophrenia. Such a hypothesis requires future clinical and basic research to assess both MAP and EXP independently, and supports the use of these clinical factors to describe negative symptoms^10^ (see ‘future directions’ in Supplementary Discussion).

Our results highlight the deleterious effect of antipsychotic-induced sedation on motivation and call for regular assessment of sedation as a potentially treatable cause of motivation deficits in patients with schizophrenia.

## Supplementary Material

Supplementary Materials

## Figures and Tables

**Figure:**
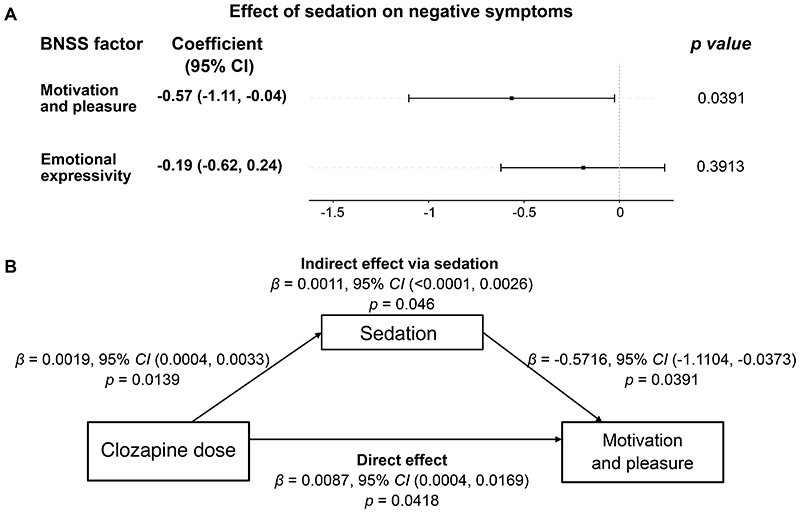


## Data Availability

Data are available from the corresponding author upon reasonable request.
