# Supplementary material for: The longitudinal effect of clozapine-associated sedation on motivation in schizophrenia
Source: Br J Psychiatry. Author manuscript; Available in PMC 2023 Jul 12. (PMC10331318; doi:10.1192/bjp.2022.191)
Supplement: Supplementary Materials [file EMS158343-supplement-Supplementary_Materials.pdf]

SUPPLEMENTARY MATERIAL

Supplementary Methods

Assessment of total hours of sleep as an index of sedation

There are several ways to measure the severity of sedation in the clinical setting. First, a clinician can rely on the patients' subjective self-reports of sedation levels. However, such reports tend to vary significantly, and do not necessarily correspond to more objective measures of drowsiness[1]. Second, one can use questionnaires measuring sleepiness and fatigue, such as the Fatigue Assessment Instrument[2], but these can be quite long for regular clinical use. They have not been designed for use in schizophrenia patients so may conflate primary and secondary 'fatigue' symptoms. Third, sedation can be assessed by a clinician using a Likert scale[3]; however, this rating is clinician-dependent and can be heavily biased. Fourth, patient daily sleeping habits can provide information about the level of sedation[4]. We have shown that the total number of hours of sleep per day (overall daytime and night-time sleep) provides a reliable measure of anti-psychotic induced sedation[4], particularly when corroborated with additional questions about sleeping habits. Estimating the average daily number of hours of sleep provides a robust approach to sleep pattern, shows high reproducibility[5] and correlates well with objective measures of sleep[6]. We showed that the total number of hours of sleep is (antipsychotic) dose-dependent within and across patients. We therefore used this measure as a clinical proxy for antipsychotic-induced (clozapine-induced) sedation.

In addition to direct questions about day and night sleep duration, the following questions were used to corroborate the patient's response: 1) How many hours do you sleep at night on average? 2) Do you have any naps during the day? 3) What time do you go to bed at night? 4) What time do you wake up in the morning? 5) Has your sleep pattern changed in the last few

weeks or months? These different questions were used to obtain a consistent estimate of total daily sleep duration. In case of a discrepancy between responses, the conflict was pointed out to the patient and questions were repeated until a consistent response was obtained. [5,6]

Study design and participants

This was a naturalistic longitudinal cohort study of clozapine-treated patients attending clinical services at Cambridgeshire and Peterborough NHS Foundation Trust, UK. The Trust provides secondary mental health services for a resident population of nearly 1 million people in a mixed urban and rural region of the East of England and is the only local provider of clozapine. All patients in the study were diagnosed with schizophrenia or schizoaffective disorder according to DSM-IV[7]. All patients started to take clozapine at least one year prior to being recruited to the study. This makes transient sedative effects less relevant, as sedation typically stabilises after six months of clozapine use (see Fernandez-Egea et al., 2021).

All patients were under the care of a senior consultant psychiatrist (EFE) who performed all relevant clinical assessments, scales, and questionnaires during routine care. All assessments were entered into the Clinical and Research Database for Persistent Schizophrenia (CRDPS). The database was approved by an NHS Research Ethics Committee (REC; references 13/EE/0121 and 18/EE/0239). This study covers information obtained from 11th December 2012 to 31st December 2021. Only assessments with a standardised evaluation of negative symptom using the Brief Negative Symptom Scale (see below) were included; this is routinely completed every two years.

All care plan assessments include relevant sociodemographic and clinical information, such as sex and age, a review and confirmation of all prescribed medication (including dose), last

medication change, smoking status (average number of cigarettes per day), alcohol use (average number of alcohol units per week). We did not assess the extent of caffeine use in patients. Importantly, however, caffeine use has a significantly smaller impact on clozapine levels than smoking[8]. Moreover, caffeine use is unlikely to lead to a consistent bias, as different people would likely consume different amount of caffeine. The absence of caffeine use in our model would therefore likely lead to a loss of power in the analyses.

Supplementary Discussion

Future directions

Although negative symptoms are highly debilitating and determine patient long-term prognosis in patients with schizophrenia, these symptoms remain poorly understood and poorly treated[9–11]. The major relevance of our findings is in establishing the relationship between clozapine dose, sedation severity, and motivation and pleasure deficits in schizophrenia, via direct and indirect pathways. We did not explore the effects of other medications on negative symptom domains as this was not within the scope of this project on sedation. Only aripiprazole was included as we have previously shown an effect on sedation[4] and due to a high prevalence in our sample (~30% cases, **Table S1**). Nevertheless, for instance, antidepressants are considered the first choice for treating negative symptoms, but with weak evidence to date[11], as studies and meta-analyses have used non-specific assessment tools for negative symptoms. We believe that the longitudinal mediation analysis shown here might provide a blueprint for future studies to examine the differential effect of medications on primary and secondary negative symptoms (and their main factors). Here we only explored sedation, but other more complex models could incorporate more than one indirect path (e.g., a mirtazapine direct effect on negative symptoms, but also an indirect effect via sedation and depression). This will require larger sample sizes of longitudinal cohorts.

Strengths and limitations

The strengths of this study include a relatively large cohort of patients, followed up in the natural setting of a UK National Health Service clinic. Moreover, we used the BNSS, which is a specific assessment tool for negative symptoms, with high content validity [12–14]. Further, we used multi-level mediation[15] to separate clozapine-related sedation from the direct effect of clozapine on motivation, as discussed in the main text. Finally, the longitudinal design allowed

assessment of the effects of changes in medication, sedation, and other clinical factors on negative symptom factors within a patient. A within-patient design allows a more robust statistical analysis and enables the separation of effects of confounding variables from the effect of interest. For example, clozapine dose is influenced by smoking status and sex, which we controlled for, but also by individual differences in drug metabolism, which we control for using the within-patient design (in contrast to a cross-sectional design, in which dose is not a reliable measure of clozapine levels).

Our study has limitations that should be considered. Although the sample size was relatively large, it was not large enough to address more specific questions about BNSS items, or the role of other medications and modifiers of negative symptoms. Confidence intervals were wide; a larger sample size is therefore required for a replication and a better estimation of effect sizes, although there is a scarcity of well-characterised clozapine-treated cohorts. An additional contributor to uncertainty in our study is likely to have been clinical variables that were not accounted for, such as medications. Importantly, however, medications and other unaccounted-for clinical variables are unlikely to have been unevenly or systematically distributed in the patient sample, so are likely to have reduced statistical power (making it more difficult to establish a significant association between clozapine and MAP and EXP) but unlikely to have caused a systematic bias in the results. Furthermore, patients were only included who had been treated with clozapine for at least 12 months, to assess “established” sedative effects. Other studies are needed to explore the sedating effect of patients treated with other antipsychotics. Moreover, approximately 80% of the patients were male. While this is a common sex distribution bias in treatment-resistant schizophrenia cohorts, we recognise this might impact the generalisability of our results. Lastly, there is currently no widely accepted method to assess sedation routinely in clinics for patients with schizophrenia. This calls for further clinical

research to look at the commonalities and differences across the different methods used for assessing sedation routinely.

Supplementary figures and tables

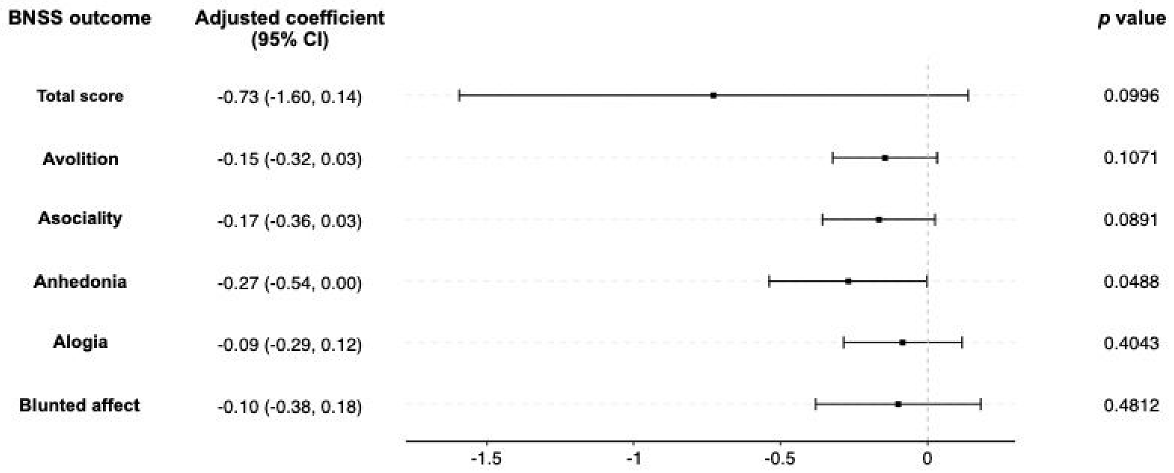

Figure S1. Association between sedation and individual symptom domains. Coefficients and their 95% confidence intervals (CI). Coefficients, CIs, and *p* values were estimated from a multi-level linear regression with sedation as the key predictor, controlling for age at baseline, sex, severity of psychosis, severity of depression, clozapine dose, aripiprazole dose, smoking status, and alcohol consumption. A random-effects intercept was fitted for each participant. BNSS, Brief Negative Symptom Scale.

**Variables**	**Number (percentage %) or mean (standard deviation)**
**Per person:**	
Age (baseline)	47.4 (10.4)
Sex (= male)	150 (80.2%)
Follow-up (months)	25.4 (21.4)
Number of face-to-face assessments	
1	53 (28.3%)
2–4	130 (69.5%)
5–10	4 (2.14%)
**Per assessment:**	
Overall sleep duration (hours per day)	9.17 (1.7)
Smoking status (= yes)	151 (37.9%)
Amount among smokers (cigarettes per day)	18.0 (9.82)
Alcohol status (= yes)	164 (40.8%)
Amount among drinkers (units/week)	15.4 (22.5)
BNSS score (total)	49.03 (16.66)
Motivation and pleasure (sum of items 1–3, 5–8)	27.89 (10.2)
Emotional expressivity (sum of items 9–13)	27.14 (7.97)
Anhedonia (sum of items 1–3)	12.24 (4.75)
Asociality (sum of items 5–6)	7.62 (3.45)
Avolition (sum of items 7–8)	8.03 (3.29)
Blunted affect (sum of items 9–11)	12.44 (5.02)
Alogia (sum of items 12–13)	8.69 (3.52)
PANSS Positive score (sum of items P1 to P7)	12.5 (4.56)
Calgary Depression Scale score	3.69 (4.12)
Duration of clozapine treatment (years)	17.86 (11.25)
Clozapine dose (mg/day)	321.42 (137.83)
Aripiprazole dose (%BNF maximum [30 mg/day])	12.31 (22.7)

Table S1. Basic demographic and clinical information of the patient sample (*n* = 187 patients, 398 assessments). BNF, British National Formulary; BNSS, Brief Negative Symptom Scale; PANSS, Positive and Negative Syndrome Scale.

Medication	Number (proportion) of patients treated
aripiprazole	59 (31.6)
risperidone	2 (1.1)
sulpride	8 (4.3)
amisulpride	20 (10.7)
quetiapine	1 (0.5)
haloperidol	2 (1.1)
olanzapine	1 (0.5)
chlorpromazine	1 (0.5)
citalopram	38 (20.3)
escitalopram	2 (1.1)
paroxetine	4 (2.1)
venlafaxine	10 (5.3)
duloxetine	2 (1.1)
fluoxetine	21 (11.2)
fluvoxamine	1 (0.5)
sertraline	14 (7.5)
mirtazapine	5 (2.7)
clomipramine	3 (1.6)
lithium	6 (3.2)
valproate	15 (8)
lamotrigine	5 (2.7)
propranolol	29 (15.5)
atenolol	2 (1.1)
zopiclone	6 (3.2)
clonazepam	5 (2.7)
lorazepam	11 (5.9)
diazepam	6 (3.2)

Table S2. Medication use in the study cohort.

Predictor	Coefficient, *β* (95% CI)	*p*
Age at baseline (years)	-0.1278 (-0.2549, -0.0007)	0.0531
Sex (male vs. female)	**-4.4051 (-7.6392, -1.1711)**	**0.0091**
Sedation (as hours of sleep per day)	**-0.5716 (-1.1104, -0.0373)**	**0.0391**
Psychosis (PANSS positive symptom score)	**-0.3810 (-0.5944, -0.1685)**	**0.0006**
Calgary depression score	**-0.5685 (−0.8074, -0.3294)**	**<0.0001**
Clozapine dose (mg/day)	**0.0087 (0.0004, 0.0169)**	**0.0418**
Aripiprazole dose (%BNF maximum)	0.0211 (-0.0259, 0.0683)	0.3856
Smoking (yes vs. no)	**-0.1100 (-0.2153, -0.0045)**	**0.0438**
Alcohol (units per week)	0.0234 (-0.0337, 0.0795)	0.4203

BNF, British National Formulary; PANSS, Positive and Negative Syndrome Scale.

Table S3. Predictors of individual differences in motivation and pleasure. Results of the linear mixed effects model estimating the predictors of motivation and pleasure (sum of items 1–3 and 5–8 in the Brief Negative Symptom Scale) across the clinical assessments longitudinally. Sex and smoking were categorical variables. Significant predictors are shown in bold.

Supplementary references

1. Klepstad P, Hilton P, Moen J, Fougner B, Borchgrevink PC, Kaasa S. Self-reports are not related to objective assessments of cognitive function and sedation in patients with cancer pain admitted to a palliative care unit. *Palliat Med*. 2002;16(6):513-519. doi:10.1191/0269216302pm587oa

2. Schwartz JE, Jandorf L, Krupp LB. The measurement of fatigue: A new instrument. *Journal of Psychosomatic Research*. 1993;37(7):753-762. doi:10.1016/0022-3999(93)90104-N

3. Fervaha G, Takeuchi H, Lee J, et al. Antipsychotics and Amotivation. *Neuropsychopharmacol*. 2015;40(6):1539-1548. doi:10.1038/npp.2015.3

4. Fernandez-Egea E, Chen S, Jenkins C, et al. The Effect of Clozapine on Self-reported Duration of Sleep and Its Interaction With 23 Other Medications: A 5-Year Naturalistic Study. *Journal of Clinical Psychopharmacology*. 2021;41(5):534-539. doi:10.1097/JCP.0000000000001432

5. Patel SR, Ayas NT, Malhotra MR, et al. A prospective study of sleep duration and mortality risk in women. *Sleep*. 2004. doi:10.1093/sleep/27.3.440

6. Gaina A, Sekine M, Chen X, Hamanishi S, Kagamimori S. Validity of child sleep diary questionnaire among junior high school children. *Journal of Epidemiology*. 2004. doi:10.2188/jea.14.1

7. American Psychiatric Association. *Diagnostic and Statistical Manual of Mental Disorders, Fourth Edition, Text Revision (DSM-IV-TR)*. Vol 1. 4th ed. Arlington, VA: American Psychiatric Association; 2000. doi:10.1176/appi.books.9780890423349

8. de Leon J. Psychopharmacology: Atypical Antipsychotic Dosing: The Effect of Smoking and Caffeine. *PS*. 2004;55(5):491-493. doi:10.1176/appi.ps.55.5.491

9. Bègue I, Kaiser S, Kirschner M. Pathophysiology of negative symptom dimensions of schizophrenia – Current developments and implications for treatment. *Neuroscience & Biobehavioral Reviews*. 2020;116:74-88. doi:10.1016/j.neubiorev.2020.06.004

10. Carpenter WT Jr, Heinrichs DW, Alphs LD. Treatment of Negative Symptoms. *Schizophrenia Bulletin*. 1985;11(3):440-452. doi:10.1093/schbul/11.3.440

11. Galderisi S, Kaiser S, Bitter I, et al. EPA guidance on treatment of negative symptoms in schizophrenia. *European Psychiatry*. 2021;64(1). doi:10.1192/j.eurpsy.2021.13

12. Mucci A, Galderisi S. The second-generation assessment scales: Brief negative symptom scale and clinical assessment interview for negative symptoms. *European Psychiatry*. 2016;33(S1):S70-S70. doi:10.1016/j.eurpsy.2016.01.980

13. Strauss GP, Keller WR, Buchanan RW, et al. Next-generation negative symptom assessment for clinical trials: Validation of the Brief Negative Symptom Scale. *Schizophrenia Research*. 2012;142(1):88-92. doi:10.1016/j.schres.2012.10.012

14. Kirkpatrick B, Strauss GP, Nguyen L, et al. The Brief Negative Symptom Scale: Psychometric Properties. *Schizophrenia Bulletin*. 2011;37(2):300-305. doi:10.1093/schbul/sbq059

15. Krull JL, MacKinnon DP. Multilevel Modeling of Individual and Group Level Mediated Effects. *Multivariate Behavioral Research*. 2001;36(2):249-277. doi:10.1207/S15327906MBR3602_06
